# Ultrafast optical circuit switching for data centers using integrated soliton microcombs

**DOI:** 10.1038/s41467-021-25841-8

**Published:** 2021-10-15

**Authors:** Arslan Sajid Raja, Sophie Lange, Maxim Karpov, Kai Shi, Xin Fu, Raphael Behrendt, Daniel Cletheroe, Anton Lukashchuk, Istvan Haller, Fotini Karinou, Benn Thomsen, Krzysztof Jozwik, Junqiu Liu, Paolo Costa, Tobias Jan Kippenberg, Hitesh Ballani

**Affiliations:** 1 Swiss Federal Institute of Technology Lausanne (EPFL), CH-1015 Lausanne, Switzerland; 2 Microsoft Research, 21 Station Road, Cambridge, CB1 2FB UK

**Keywords:** Fibre optics and optical communications, Optoelectronic devices and components, Frequency combs, Solitons

## Abstract

Due to the slowdown of Moore’s law, it will become increasingly challenging to efficiently scale the network in current data centers utilizing electrical packet switches as data rates grow. Optical circuit switches (OCS) represent an appealing option to overcome this issue by eliminating the need for expensive and power-hungry transceivers and electrical switches in the core of the network. In particular, optical switches based on tunable lasers and arrayed waveguide grating routers are quite promising due to the use of a passive core, which increases fault tolerance and reduces management overhead. Such an OCS-network can offer high bandwidth, low network latency and an energy-efficient and scalable data center network. To support dynamic data center workloads efficiently, however, it is critical to switch between wavelengths at nanosecond (ns) timescales. Here we demonstrate ultrafast OCS based on a microcomb and semiconductor optical amplifiers (SOAs). Using a photonic integrated Si_3_N_4_ microcomb, sub-ns (<520 ps) switching along with the 25-Gbps non-return-to-zero (NRZ) and 50-Gbps four-level pulse amplitude modulation (PAM-4) burst mode data transmission is achieved. Further, we use a photonic integrated circuit comprising an Indium phosphide based SOA array and an arrayed waveguide grating to show sub-ns switching (<900 ps) along with 25-Gbps NRZ burst mode transmission providing a path towards a more scalable and energy-efficient wavelength-switched network for data centers in the post Moore’s Law era.

## Abstract (web summary)

Optical technologies could enable fast and power-efficient networks for data centers. Here, the authors report Si_3_N_4_ microcomb based ultrafast photonic switching to provide enhanced performance for data center applications.

## Introduction

The recent growth in data center traffic due to emerging applications such as machine learning, resource disaggregation, and large-scale data analytics, poses tight requirements on the network in terms of low latency, high bandwidth, and high scalability. Current data center networks comprise a multi-tier fabric of electrical switches and optical transceivers that consume a significant amount of power and this is expected to exacerbate due to the slowdown of Moore’s law^1,2^.

Optical circuit switching (OCS) has been proposed as an alternative technology to overcome these challenges; it can provide high bandwidth and low network latency (due to the lack of buffers in the network core), and by allowing for a flat data center topology, the network can be more energy-efficient as fewer electrical switches and transceivers are required^3^. In OCS the servers are optically interconnected directly without needing the opto-electronic-opto (O-E-O) conversion. Micro-electro-mechanical (MEMS) based OCS systems are commercially available with the possibility of high port counts (~1000 ports)^4,5^. However, MEMS-based OCS architectures suffer from slow switching time (~ms), which can result in severe bandwidth under-utilization. Some emerging workloads are characterized by very small packets, e.g., in a recent key-value store application, more than 97% of the packets have a size of 576 bytes or less^6^. Assuming today’s 50-Gbps speeds, it takes <93 ns to transmit a 576-byte packet. Therefore, achieving a reconfiguration overhead lower than 10% would require a nanosecond switching time.

Many photonic chip-based techniques have been used to demonstrate the fast optical switching (ns) such as semiconductor optical amplifiers (SOAs)^7^, electro-absorption modulators (EAMs)^8^, and Mach–Zehnder interferometers (MZIs)^9,10^. The arrayed waveguide grating routers (AWGRs)^11,12^, in conjunction with a tunable laser (TL), is a promising OCS technology as the core of the network is passive. This improves fault tolerance and it is also beneficial for future scaling because, unlike with today’s electrical switches, the core may not need to be upgraded when data rates increase. However, state-of-the-art tunable lasers with custom drivers can have a tuning latency of 10 ns or higher^13,14^ while custom lasers and drivers have been combined to achieve a latency of around 5 ns^15,16^, which make them unsuitable for our targets. A recent proposal addresses this issue by showing that using a disaggregated laser architecture in which the wavelength generation is decoupled from the wavelength selection, it is possible to achieve sub-nanosecond laser tuning time^17,18^. One possible implementation of this architecture relies on a discrete bank of lasers as a multi-wavelength source and a wavelength selector containing photonic integrated arrayed waveguide grating (AWG) and SOAs. The SOAs are preferred as switching elements due to fast and lossless switching, chip-scale integration, and high extinction ratio (ER)^7^.

**Fig. 1:**
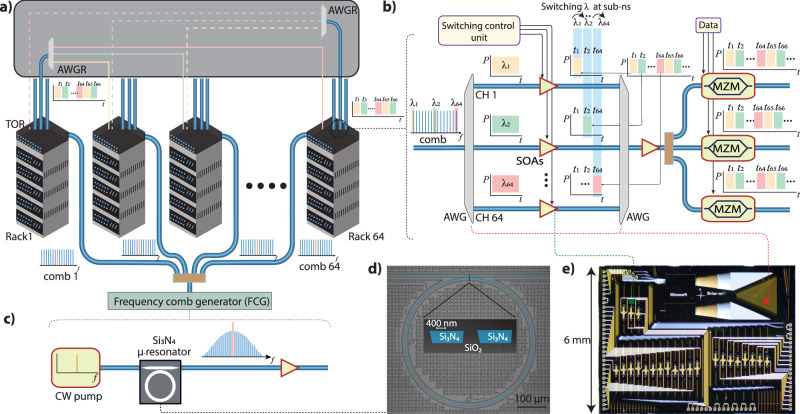
Concept of optical circuit switching (OCS) using a photonic chip-based **Si**_3_**N**_**4**_ soliton microcomb and semiconductor optical amplifiers (SOAs). **a** Interconnection of 64 racks via an arrayed waveguide grating router (AWGR) for implementing fast OCS. In this model, distinct wavelengths are assigned to each rack at each time slot. At each receiver, a 10–20 nanosecond (ns) time slot is assigned for each rack on a round-robin basis for data transmission. The switching module is placed on the top of rack (TOR) switch. A single comb source that is post amplified via cascaded amplifiers to attain high optical power per line can be distributed among many racks. The 64 individual combs (comb1, ..., comb 64) split from a central frequency comb generator (FCG) can be distributed across 64 different racks as a multiwavelength laser, making this architecture more power-efficient and flexible. The multiple data-carrying optical carriers are routed using a passive AWGR to the assigned racks. **b** Each comb channel is transmitted to SOAs after de-multiplexing, where a control signal (turning on/off current) is applied to switch between the comb channels at sub-ns. The comb channels (10-ns slots) are encoded with data using Mach-Zehnder modulators (MZMs) and transmitted to the relevant racks. The multiple MZMs shown here indicate that this architecture can be scaled further to establish links between more racks. **c** The multi-wavelength source based on the chip-scale soliton microcomb is generated by pumping with a single laser. Microscope images of a Si_3_N_4_ microresonator (**d**) and a photonic chip (**e**) containing an AWG and SOAs. The inset in (**d**) shows a false color SEM image of a Si_3_N_4_ microresonator’s coupling section.

In this paper we propose and experimentally demonstrate a disaggregated tunable transceiver that uses photonic chip-based soliton microcombs as a multi-wavelength source, which are generated in high-quality (*Q*) microresonators exhibiting third-order nonlinearity (*χ*^3^) and anomalous dispersion^19,20^. The soliton microcombs have been used in many system-level applications, for example, distance ranging (LiDAR)^21–23^, microwave photonics^24–27^, optical coherence tomography (OCT)^28,29^, and coherent communication^30–32^. The remarkable improvements in the losses of high-confinement Si_3_N_4_ integrated waveguides, a widely used platform for integrated photonics^33,34^, allows the generation of solitons using the on-chip laser^35–37^. The broadband-bandwidth operation across C- and L-bands, precise spacing control to match ITU frequency grid (50/100 GHz), low power consumption, and wafer-scale scalability make the soliton microcomb an attractive solution for an ultra-fast wavelength switched system in comparison with a bank of lasers^30^. Further, no extra guard band is required for frequency stabilization or to align each comb channel to the frequency grid. A key advantage of the disaggregated transceiver design is that the multi-wavelength comb source can be shared across many (e.g. 64) nodes (racks or servers) in parallel instead of using 64 separate comb sources, using a split-and-amplify architecture (Fig. 1a). The source can thus be treated as a shared infrastructure element such as the power source in today’s data centers. This allows for an appealing division of functionality since the power consumed by the comb source is amortized as the power efficiency of the end-to-end system converges to the efficiency of the amplifiers while allowing for a high-quality and stable light source that can be rapidly wavelength-tuned (cf. discussion on power analysis and Supplementary information (SI)).

Here, we use a Si_3_N_4_ based soliton microcomb as a multi-wavelength source to show ultrafast optical wavelength switching. In a proof-of-concept experiment, we demonstrate switching of >20 individual comb channels at sub-ns time scale (<520 ps) using discrete SOAs. Further, 25-GBd (baud-rate) non-return-to-zero on-off keying (NRZ) and four-level pulse amplitude modulation (PAM-4) burst mode transmission systems along with ultrafast switching are shown. Then, a more compact switching system consisting of PIC-based AWG and SOAs is implemented to show sub-ns switching and 25-GBd NRZ burst mode transmission, indicating the potential utilization of such a miniaturized system to mitigate power and scaling issues.

## Results

### OCS architecture based on soliton microcomb

**Fig. 2:**
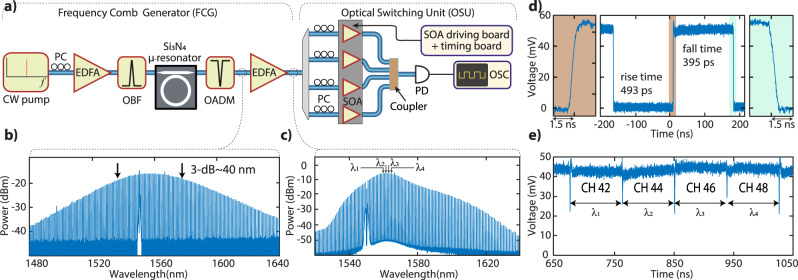
Experimental demonstration of sub-ns optical circuit switching (OCS) of single and four different microcomb channels. **a** A packaged Si_3_N_4_ microresonator is pumped using a continuous wave (CW) pump amplified via an erbium-doped fiber amplifier (EDFA) to generate a soliton microcomb. A soliton state is initiated via forward tuning. After filtering the pump using an optical add-drop multiplexer (OADM), a low-noise amplifier is used to further amplify the soliton comb. The post-amplified comb is de-multiplexed using a 48-channels 100G AWG, and the individual comb channels are routed to semiconductor amplifiers (SOAs) which are controlled via a custom-designed switching board. The output signal is detected using a photodiode (PD) and recorded on an oscilloscope (OSC). PC polarization controller, OBF (narrow) optical bandpass filter. **b** A single soliton spectrum with 3 dB bandwidth around 40 nm and no post amplification. **c** Post-amplified soliton spectrum having maximum power up to −4 dBm in comb lines close to pump line (1550 nm). **d** The 10%–90% rise and fall time of 493 and 395 ps respectively for single comb channel (CH 37: 1554.9 nm). The left and right insets show the zoomed-in view of rising and falling signals. **e** The four different microcomb channels simultaneously switching at sub-ns time scale with wavelength separation ~4.9  nm. A guard zone of ~2.56 ns is used to allow smooth switching between two adjacent channels. The ER is lower in comparison with (**d**) as signal intensity does not drop to zero while switching between adjacent channels (see “Methods”, Switching control unit). CH-42: 1558.988 nm, CH-44: 1560.613 nm, CH-46: 1562.238 nm, and CH-48: 1563.863 nm.

Figure 1 shows the OCS architecture containing the soliton microcomb, SOAs, AWGs in the switch and AWGRs to route the data across many racks (cf. Methods). This architecture allows further parallelization of different resources by sharing the soliton across many racks and modulators for power efficiency and parallel data transmission, respectively (Fig. 1a, b). The multi-wavelength source is generated by pumping a packaged Si_3_N_4_ microresonator fabricated using the photonic Damascene reflow^38,39^ process enabling a mean intrinsic *Q*-factor (*Q*_0_) of >15 million. Initially, a multi-soliton is initiated by performing a scan over resonance (forward tuning)^20^ and then a single soliton is generated via backwards switching^40^ (Fig. 2b). The soliton is amplified using a low-noise and compact EDFA resulting in a comb with a maximum power of up to −4 dBm and an optical signal-to-noise ratio (OSNR) of >34 dB. The post-amplified soliton as shown in Fig. 2c is de-multiplexed using a 100G spaced 48-channel AWG (~1525–1564 nm) providing 30 dB isolation. The individual comb channels are initially switched using discrete SOAs with a small-signal gain of ~11–13 dB at 1550 nm. Figure 2d shows 10–90% rise and fall times of 493 and 395 ps, respectively, for a single microcomb carrier centered at 1555 nm (CH 37 of AWG) when applying a current of 120 mA to operate the SOA. Similarly, more than 20 comb channels (1540–1564 nm) in C-band are tested individually to show sub-ns switching (cf. SI). Even though not tested due to the unavailability of an L-band AWG, the current results indicate that more than 40 comb channels in the L-band could be used as well.

By scaling to thousands of nodes, we can take advantage of the fact that today’s servers or top-of-the-rack (ToR) switches comprise multiple channels. For example, the recently announced NVIDIA Ampere A100 GPU^41^, supports 2.4 Tbps of bandwidth by combining 48 channels, each operating at 50 Gbps. Therefore, assuming 40 wavelengths and SOAs per transmitter, we can connect up to 48 × 40 = 1920 other nodes. If, instead, we consider a rack-based deployment, the latest ToR switches^42^ have 512 SERDESes (i.e., 256 uplinks) that could allow to interconnect up to 256 × 40 = 25,600 racks, which is an order of magnitude higher than the number of racks in even large data centers today. However, this also means that a node is directly connected to any other node in the data center through only one of its uplink channels. To ensure that any pair of nodes can still communicate with their full bandwidth, the network routes traffic between any pair of nodes through all other nodes in the network. Such detour routing imposes a throughput overhead although the throughput can be at most 2× worse than an ideal switch and thus, can be compensated by doubling the per-node network bandwidth. Furthermore, detour routing offers several advantages including the fact that it obviates the need for explicitly scheduling network traffic, which has been a key bottleneck for practical and deployable optical switching in data center. We provide more details of our network architecture and the trade-offs imposed by our topology and routing choice in a separate paper^18^.

### Ultra-fast wavelength switching and data transmission via discrete SOAs

For a proof-of-concept system-level demonstration, fast switching within four different comb channels is performed. Figure 2e shows sub-ns switching between four different comb channels with a wavelength separation of ~5.6 nm. A guard zone of 2.56 ns is used to allow a smooth switching between adjacent comb channels while an external reference clock (timing board) aligns the different switching signals. Distinct currents are applied to the SOAs to achieve a constant output power and to compensate for the non-uniform comb power per line or SOA’s gain (Fig. 2e). While the sub-ns switching of four channels with a maximum 20 nm separation has been demonstrated, the maximum channel separation is mainly limited by the optical band-pass filter (OBF) (cf. SI).

**Fig. 3:**
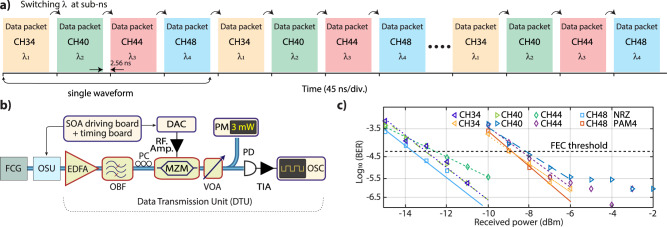
Experimental demonstration of burst mode NRZ and PAM-4 transmission using discrete SOAs while switching. **a** A stream of multiple data packets showing data transmission along with sequential switching between the four comb channels. A single burst waveform sequence consists of header, payload, and guard zone containing 32, 1024, and 64 symbols respectively. **b** The signal after the frequency comb generator (FCG) and the optical switching unit (OSU) is amplified using a compact EDFA to compensate for losses of the 20-GHz Mach-Zehnder modulator (MZM). Then, it is filtered out using a wide-band optical bandpass filter (OBF) (~20 nm) to reject amplified spontaneous emission (ASE) noise from the SOAs and the EDFA. The data are encoded on the modulator using an arbitrary waveform generator (DAC). A fast photodiode (PD) is used to detect the signal. The electrical signal is amplified using a trans-impedance amplifier (TIA) and finally captured by an OSC. **c**) The bit error ratio (BER) of four different comb channels while switching between them, using different modulation formats non-return to zero (NRZ) and four-level pulse amplitude modulation (PAM-4). A performance below forward error correction (FEC) threshold is achieved for NRZ and PAM-4 for a received optical power of ~−12 dBm and ~−8 dBm, respectively. CH-34: 1552.524 nm, CH-40: 1557.363 nm, CH-44: 1560.606 nm, and CH-48: 1563.863 nm.

In the following experiment, we show 25 Gbps (NRZ) and 50 Gbps (PAM-4) burst mode data transmission while switching between four comb channels using the setup shown in Fig. 3b. The four optical carriers after switching are further amplified to overcome the insertion loss (~7 dB) of the 20-GHz Mach-Zehnder modulator (MZM) which is operating at the quadrature point. In addition to eliminating comb channels in the next order FSR of the AWG, the OBF is utilized to suppress the out-of-band SOA and EDFA amplified spontaneous emission (ASE) noise. The burst mode sequence at 25 GBaud symbol rate, generated by the arbitrary waveform generator is applied to the MZM with a random sequence of 2^15^- and 2^16^-bits for NRZ and PAM-4 respectively. The electrical waveform is amplified using a trans-impedance amplifier (TIA) after detecting it on a fast photodiode having 50-GHz bandwidth. The amplified waveform is acquired using a real-time oscilloscope with 160 GSamples/s sampling rate. The digital signal processing (DSP), explained in detail in ref. ^17^, is performed offline to obtain the bit error ratio (BER). The received optical power (ROP) vs. (BER) of the system, as shown in Fig. 3c, is characterized by changing the optical power of incoming waveform via a variable optical attenuator (VOA). A BER of below 5 × 10^−5^, which is the threshold for forward error correction (FEC) in data center transmission systems corresponding to the KP4 FEC^43^, is achieved for both NRZ and PAM-4 at a ROP of ~−12 dBm and ~−8 dBm respectively. The BER error floor for PAM4 at a ROP of >−6 dBm emerges due to ASE and AWG crosstalk.

### Photonic integrated circuit based optical switching and data transmission

**Fig. 4:**
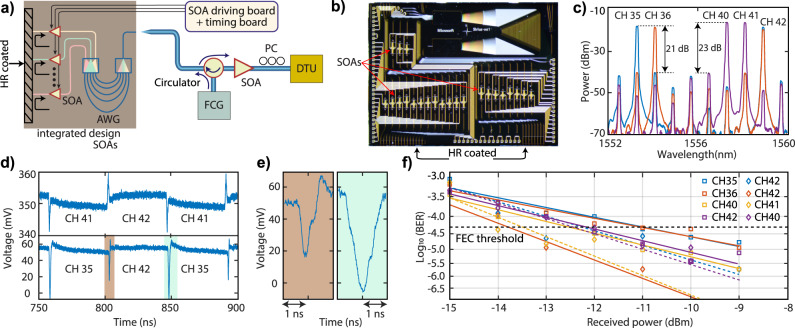
Sub-ns optical circuit switching (OCS) and data transmission using on-chip SOAs and on-chip AWG along with soliton microcomb. **a** Schematic of the setup used to perform the OCS and data transmission. The multi-wavelength optical carriers, generated via the frequency comb generator (FCG), are coupled to an InP chip containing an AWG and SOAs via an optical circulator. The coupled optical carriers are aligned to the AWG by changing the temperature of InP chip. The aligned carriers are transmitted to integrated SOAs; if one of them is biased, then the AWG channel (waveguide) connected to that particular SOA is reflected from the high-reflection coated facet of the chip while non-biased SOAs block the light. The reflected-back channels are coupled back via an anti-reflection coated optical fiber for encoding the information using the data transmission unit (DTU). **b** Microscope image of PIC showing the SOAs (red arrows) and AWG. **c** Optical spectrum of comb channels with different spacing while switching after the InP chip indicating more than 20 dB isolation with adjacent AWG channels (cross talk). The red curve shows CH 36 and 42 with 4.8 nm wavelength spacing, the blue curve CH 35 and 42 (5.6 nm) and the purple curve CH 40 and 41 (0.8 nm). **d** The sub-ns wavelength switching between two different comb channels using on-chip SOAs. The overshoot in the switching signal is due to impedance mismatch between the high-speed radio-frequency (RF) probes and the on-chip electrodes. This effect can be minimized by optimizing the drive signal. **e** The left and right figures show the zoomed-in view of switching signals between two different comb channels (CH35 and CH 42). **f** The bit error ratio (BER) performance of the 25 GBd NRZ PIC-based switching system for different combinations of two comb channels.

Next, integrated Indium phosphide (InP) chip-based SOAs and AWG are used to show 25-Gbps NRZ burst mode data transmission along with fast OCS using a soliton microcomb. Figure 4b shows the photonic integrated circuit (PIC) based wavelength selector PIC with a dimension of 6 mm × 8 mm. The reflection of the light from the high-reflection (HR) coated facet allows simultaneous utilization of an AWG with 32 × 50 GHz separated channels as multiplexer and de-multiplexer. This simplifies the wavelength alignment procedure and reduces the footprint of the device. Nineteen SOAs are connected via integrated waveguides to AWG output channels. The wavelength alignment of the comb channels to the AWG is performed by changing the temperature of the PIC, resulting in seven comb channels matching with the AWG. This can also be realized by changing the temperature of the Si_3_N_4_ chip. Figure 4c shows the optical spectrum of the AWG aligned comb channels indicating a >20 dB isolation with adjacent channels. Initially, the 10–90% rising and falling times of PIC is characterized by performing simultaneous switching between two comb channels. The maximum (minimum) experimentally observed switching time is ~820 ps (~375 ps). Moreover, the overshoot in the switching signal, as seen in Fig. 4d, arises due to impedance mismatch between the SOAs on-chip electrodes and the RF probes^17^. Then, a burst mode data transmission demonstration with 25-Gbps NRZ modulation is performed. A BER below FEC threshold is obtained when switching between two-channels with different combinations for a ROP >−11 dBm. While channels 40 and 41 have approximately the same optical power, channel 41 shows better BER performance as the crosstalk from channel 41 to 40 is about 8 dB lower than the crosstalk from channel 40 to 41. The PAM-4 burst mode transmission demonstration requires further improvement in the output power of the comb due to low in- and out-coupling in a packaged Si_3_N_4_. The main reason behind the low coupling efficiency (15%) is an additional 2 dB splicing loss between UHNA and SMF-28 fiber, which can be reduced to <0.2 dB by using state-of-art splicing instruments^44^. Similarly, the AWG crosstalk and insertion loss of the PIC can be further improved along with the utilization of lower FSR microcombs (25/50 GHz) to enhance the overall performance of this architecture. Nevertheless, the current results show the potential of a soliton microcomb as a suitable multi-wavelength source for 25 GBd NRZ burst mode transmission along with fast switching.

## Discussion

Regarding the power consumption, the current multi-wavelength source consumes a total electrical power of ~30 W (cf. SI) providing more than 60 carriers, having an optical power >−20 dBm (~500 mW electrical power per carrier). The electrical power consumption can be improved down to <193 mW per carrier (15.5 W total) by reducing the splicing loss between UHNA and SMF fibers^44^, implementing on-chip actuators^45^ instead of a bulk temperature controller and using a power-efficient, compact distributed feedback (DFB) laser as CW pump^35,37^. By optimizing the microresonator dispersion design, it is possible to generate 122 comb channels having an optical power >−14 dBm without needing any post-amplification^30^. Furthermore, an amplifier configuration with a separate amplifier for the C- and L-band would give a comb with an optical power per line ~13 dBm as mentioned in SI of ref. ^30^.

More importantly, this high power comb source can be shared among multiple racks by adding a hierarchy of passive optical splitters and amplifiers for better power and resource utilization (Fig. 1a). The soliton microcomb source distributed among 32 racks provides carriers with P_opt_ ~ −4 dBm while consuming ~2.57 W (1.115 W) electrical power per rack by using a state of the art commercial EDFA (on-chip amplifier^46^) making it a highly power-efficient and flexible solution for the data center (cf. SI). More broadly, the flexibility of sharing the comb across many racks, with the fast wavelength selection done on the rack itself, means that the overall electrical power efficiency per channel approaches the power efficiency of the EDFAs with the comb power as only a small contributor. This indicates that an optimized shared comb source would consume a comparable electrical power to other multi-wavelength source solutions, including recent techniques that use a bank of tunable lasers as a multi-wavelength source^18,47^. Since the wavelength tuning is done on the bank of tunable lasers itself, sharing it between multiple racks would lead to an increased complexity when synchronizing the wavelength switching between the racks, due to the varying time delays between the bank of tunable lasers and the racks. As a matter of fact, one tunable laser bank per rack would be more optimal for a time-multiplexed solution^47^ which would require at least 2 × 32 tunable lasers for switching between 32 racks instead of a single amplified comb chip. Moreover, a comb does not require additional complex algorithms for fast switching and wavelength stabilization, thus offering an appealing division of functionality by leveraging a complex yet highly shared light source.

In this work, we demonstrate the possibility of achieving nanosecond OCS using a chip-based microcomb for future power-efficient and low latency data centers. More than twenty individual comb channels in C-band having a power >−20 dBm are switched at <520 ps using discrete SOAs. The OCS system with 25-GBd NRZ and PAM-4 burst mode data transmission is shown while switching between different comb channels. Further, a PIC containing on-chip SOAs and an AWG is implemented to show sub-ns switching (<900 ps) and 25-GBd NRZ transmission. The current demonstration can provide a route for a fully integrated, fast-tunable transceiver providing dense carriers for wavelength switching to meet the power and latency requirements posed by future cloud workloads.

## Methods

### Switching architecture

The link between two racks is only established via a single wavelength (comb tooth) in specific time slots (*t*_64_, *t*_128_, . . . ) as shown in Fig. 1a. The soliton microcombs provide many coherent optical carriers assigned to distinct racks. The switching operation is performed by applying a control signal on the SOAs, e.g., the switching from *t*_1_ to *t*_2_ data slot is done by applying an on signal to the second SOA and off signals to all other SOAs. A trigger signal from an external reference clock is used to align the switching control and data-encoding units.

### **Si**_3_**N**_**4**_ microresonator

The Si_3_N_4_ microresonators are fabricated by using photonic Damascene reflow process enabling waveguides with ultra-smooth sidewalls and linear propagation loss ~1 dB\m^27,38^. The designed microresonators are over-coupled (*κ*_*ex*_ ~ 4 × *κ*_0_) with a FSR of 99.5 GHz and intrinsic linewidth of 15 MHz^39^. The waveguides have a dimension of 1500 × 900 nm^2^. The double inverse nano tapers are used to facilitate the light coupling in- and out- of the chip^48^.

### Soliton microcomb generation

A compact fiber laser (Koheras BASIK) is amplified using an EDFA. Then, ASE noise is filtered out using a narrow optical bandpass filter. The Si_3_N_4_ chip is packaged by splicing the ultrahigh numerical aperture (UHNA) fiber with standard SMF-28 fiber with chip through (fiber-chip-fiber) 15% coupling efficiency^49^. A single soliton is initiated at an input power of ~450 mW in the bus waveguide by applying a custom-designed ramp voltage. The deterministic soliton initiation and backward tuning are controlled via a computer interface. The strong pump line is filtered out using an OADM. Then the soliton is amplified using a low-noise EDFA before de-multiplexing. The power conversion from CW-pump to single soliton is ~2% in the current device. An intrinsically coherent single soliton state is preferred due to low line to line power variation in 3 dB bandwidth (smooth spectral).

### Photonic integrated SOAs and AWG

The InP-based wavelength selector PIC incorporates 23 SOAs of which 4 are used as references. The other 19 SOAs are connected to an 1 × 32 AWG which acts as a multiplexer and demultiplexer. One of the PIC facets is high-reflection coated so that the light is reflected back through the SOAs and AWG to the input waveguide. The reflective single AWG design reduces the footprint compared to using two AWGs and avoids wavelength misalignment of the AWGs. The wavelength selector PIC was designed using the JEPPIX foundry and fabricated at Fraunhofer HHI.

### Switching control unit

The switching control unit supplies the bias currents and electrical switching signals to the SOAs. A negative voltage is applied to drive the SOAs at the zero-level. The current flowing through the SOAs at the zero-level was around 1 μA. To optimize the switching between two different channels, the upcoming channel starts switching on while the current channel is still switching off. The ER appears to be degraded since the signal intensity shows the sum of the channels and does not drop to the zero-level during the switching event. The ER of the waveform in Fig. 2d is ~15 dB. It is difficult to estimate actual ER as the signal was measured using a sampling scope and zero level of signal is buried in noise. This unit also controls the clock and time synchronisation of the switching signals. A time synchronization having <100 ps accuracy is achieved in current study^18^.

## Supplementary information

Supplementary Information

## Data Availability

The data used to produce the plots within this paper are available at 10.5281/zenodo.4588562.

## Code availability

The code used to produce the plots within this paper are available at 10.5281/zenodo.4588562.
