# Supplementary material for: Ultrafast optical circuit switching for data centers using integrated soliton microcombs
Source: Nat Commun. 2021 Oct 15;12:5867. doi: 10.1038/s41467-021-25841-8 (PMC8520010; doi:10.1038/s41467-021-25841-8)
Supplement: Supplementary file 1 — Supplementary Information [file 41467_2021_25841_MOESM1_ESM.pdf]

Supplementary Information to: Ultrafast optical circuit switching for data centers using integrated soliton microcombs

A. S. Raja,[1,*] S. Lange,[2,*] M. Karpov,[1,*] K. Shi,[2,*] X. Fu,[1] R. Behrendt,[2] D. Cletheroe,[2] A. Lukashchuk,[1] I. Haller,[2] F. Karinou,[2] B. Thomsen,[2] K. Jozwik,[2] J. Liu,[1] P. Costa,[2] T. J. Kippenberg,[1,†] and H. Ballani[2,‡]

[1]*Institute of Physics, Swiss Federal Institute of Technology Lausanne (EPFL), CH-1015 Lausanne, Switzerland*
[2]*Microsoft Research, 21 Station Road, Cambridge, CB1 2FB, U.K.*

I. SUPPLEMENTARY NOTE 1: SOLITON CHARACTERIZATION AND INDIVIDUAL CHANNELS SWITCHING

Supplementary figure 1 a) shows the absolute power and optical signal to noise ratio (OSNR) of the individual combs lines after post-amplification. Around 25 channels in the C-band have a power of more than -18 dBm and an OSNR of 34 dB. The four channels around the pump mode centered at 1550 nm (CH 32) have a lower OSNR due to residual ASE noise. This can be avoided either by using a narrower optical bandpass filter or by using the drop-port of the microresonators to couple out the soliton instead of the through port. All these individual comb channels are used to perform the optical circuit switching using discrete SOAs with the setup shown in figure 2a (main text). The 10%-90% rise and fall times are less than 520 ps. These results indicate that more than 40 channels in L-band can be implemented using the current OCS architecture.

II. SUPPLEMENTARY NOTE 2: FOUR COMB CHANNELS SWITCHING WITH 20-NM SEPARATION

The sub-ns switching between four different comb channels having a maximum separation of more than 20 nm is shown in supplementary figure 2. The variations in comb channel power and SOAs gain are compensated by applying different biasing currents to each SOA. A biasing current of 94 mA, 109 mA, 139 mA, and 134 mA is applied to the SOAs connected to AWG channels 35, 43, 19, and 27, respectively.

III. SUPPLEMENTARY NOTE 3: SOLITON MICROCOMB POWER CONSUMPTION

In this section, a detailed power consumption analysis is carried out to understand the system performance and to provide possible ways to improve it further. The multi-wavelength soliton microcomb is generated using the setup shown in figure 2a (main text). A compact KOHERAS BASIK fiber laser is used to pump the microresonator, which consumes approximately 1.5 W electrical power when operated at low optical power ($\sim$ 1 mW). A high power BKtel erbium-doped optical amplifier (EDFA) providing gain of up to 34 dB is used mainly to overcome the coupling losses. A single soliton is accessed at a power 1.2 W at the input fiber (450 mW in bus waveguide), while a high power EDFA operated at a power of 1.8 W compensates the additional 1.5 dB insertion loss (IL) of a narrow band optical bandpass filter. The high power EDFA consumes around 24 W of electrical power. A thermoelectric cooler (TEC) based control loop is implemented to stabilize the temperature, actuating on the Si_3N_4 chip and consuming $\sim$ 0.5 W of electrical power. After filtering out the pump, the soliton is further amplified using a low noise EDFA which consumes 4 W electrical power. The total electrical power consumed by the multi-wavelength source is 30 W and the electrical power consumed by each carrier is around 500 mW (60 carriers in C- and L-bands suitable for switching and data transmission).

Supplementary Table 1: Electrical power consumption (current system)

Components	Power consumed
Laser	1.5 W
TEC	0.5 W
High power EDFA	24 W
Low-noise EDFA (post amplification)	4 W
Total	30 W

As mentioned in the main text, the main origin of excess power consumption is the lower coupling efficiency into the packaged Si_3N_4 microresonator arising due to splicing loss between the ultra-high numerical aperture (UHNA) and SMF-28 fibers (1 dB per facet). The coupling efficiency can be improved $>$ 50% by using an optimized mode converter and by reducing the splicing loss between the UHNA-SMF fibers[1, 2]. These improvements allow the generation of solitons at an input power of 0.8 W, including 1-dB loss from the optical bandpass filter. In addition, a more compact, power-efficient (electrical power $\sim$ 0.5 W), and on-chip laser can be used to generate the soliton microcomb without requiring lasers with a stabilized module [3, 4]. Similarly, a monolithic piezoelectric or an integrated heater element can be used to stabilize the soliton, ensuring long term operation and reducing the power consumption to the range of nW to few-mW (TEC, 0.5 W) [5, 6]. These improvements, such as using an on-chip laser, optimized coupling and on-chip actuation, will reduce the power consumption of the system down to 15.5 W (193 mW per carrier). Also, it will give

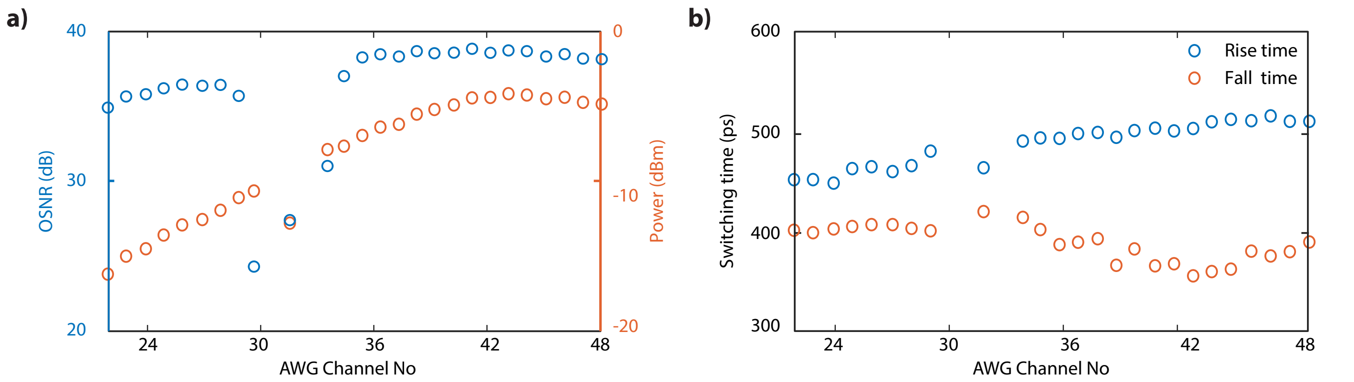

Supplementary Figure 1. **The power, optical signal to noise ratio (OSNR), and individual channel switching characterization of soliton microcomb. a)** The optical power of different channels in C-band up to -4 dBm. The OSNR of two channels around CH 32 (pump mode, ~ 1550 nm) is degraded due to ASE noise. The remaining 23 channels have an OSNR of > 34 dB. **b)** The 10%-90 % rise and fall times of around ~ 24 channels lying in the C-band with a maximum switching time of less than 520 ps while using discrete semiconductor optical amplifiers (SOAs).

access to more than 80 optical carriers as output coupling will be increased around two times. The optimization of the microresonators' different design parameters (as shown in supplementary information of ref[7]) such as coupling strength (κ_{ex}) and second-order dispersion (D_2) eliminates the need for post-amplification of comb lines. This will reduce the power consumption down to 13 W for more than 114 carriers in the C and L bands.

Supplementary Table 2:
Electrical power consumption ($P_{opt.}$ ~ 13 dBm /line)

Components	Power consumed
Laser (DFB)	0.5 W
High power EDFA	11 W
Low-noise EDFA (post amplification , C- and L-bands)	8 W
High power EDFA (post amplification , C- and L-bands)	63 W
Total	82.5 W
Per server (sharing between 32)	2.57 W

Besides, the comb source can be shared across many servers or transceivers as a parallel multi-wavelength source. As pointed in ref. [7] supplementary information, it is possible to generate the soliton microcomb in C and L-band with ~ 13 dbm power per carrier while using two additional C- and L-band amplifiers. This particular source can be distributed among 32 servers using a 1×32 splitter with an average insertion loss of around 17 dB. Each server will have a soliton microcomb source with -4 dBm power per carrier for independent wavelength switching. The total electrical power consumed by the whole system and each server is around 82.5 W and 2.57 W respectively. The power efficiency can be improved to 37 W for the whole system and 1.15 W for each server by using the on-chip amplifier[8]. This makes micro-comb based wavelength sources a more flexible and power-efficient solution for optical circuit switching in the data center.

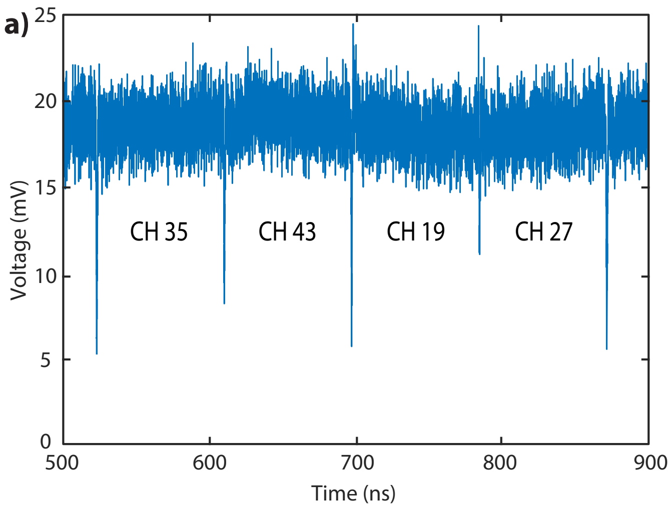

Supplementary Figure 2. **The fast wavelength switching between four different comb channels spanning 20 nm. a)** The simultaneous switching of four different comb channels using discrete SOAs. The limitation of the wavelength span in the current system is due to an optical bandpass filter used to reject channels in the next order of free spectral range from C-band AWG.

Next, we evaluated the OSNR of a single comb generator shared between 32 nodes while a single node provides 4x122 individual connections (supplementary figure 3). The following analytical expressions are used to estimate the OSNR.

$$OSNR = \frac{P_{out}}{P_{ASE}}, \tag{1}$$

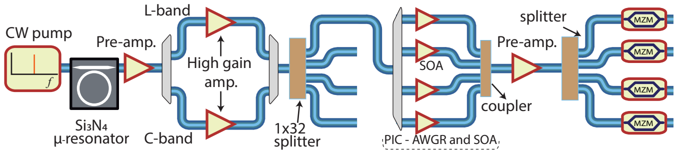

Supplementary Figure 3. **The experimental setup to generate the comb lines having 13 dBm to shared across multiple nodes and transmitters.** The soliton microcomb after the Si_3N_4 chip is amplified using a low noise pre-amplifier ($G \sim$ 12dB, F(noise figure) $\sim 4.5 - 5$) [9]. The pre-amplified microcomb is separated into C and L bands via demux (Insertion loss (I.L.) $\sim$ 0.5 dB) and then amplified using high gain amplifiers ($G \sim 20 - 25$dB, F(noise figure) $\sim 5.5 - 6$) [9]. The post amplified soliton is further split into 32 channels using a 1x32 splitter (I.L. $\sim$ 17 dB). One of the split channels is transmitted through AWGR (I.L. $\sim$ 4 - 6 dB) and SOAs ($G \sim$ 10dB, F(noise figure) $\sim$ 6) for fast optical wavelength switching[10]. The signal is further amplified using a low noise amplifier ($G \sim$ 12dB, F(noise figure) $\sim 4.5 - 5$) after switching to shared across multiple transmitters (MZM).

where P_{out} and P_{ASE} are the output power and the ASE noise of a single comb channel after being amplified by an amplifier or a SOA. The amplified output power of each comb channel after EDFA is given by $P_{out} = GP_{comb}$, where G is the gain. The ASE noise power is given by $P_{ASE} = h\nu B_{ref}(FG - 1)$ and h is the Planck constant, ν is the optical frequency of comb channel, F is the noise figure of an EDFA and B_{ref} is the reference bandwidth for noise measurement (12.5 GHz). The total gain (G) and noise figure (F) of the system consisting of a chain of amplifiers is given by

$$G_t = \prod_{n=1}^{N} \alpha_n G_n \tag{2}$$

$$F_t = \frac{1}{G_t} + \left(F_1 + \frac{F_2}{G_1\alpha_1} + \frac{F_3}{G_1\alpha_1 G_2\alpha_2} + ... + \frac{F_n}{\prod_{n=1}^{N-1} \alpha_n G_n} \right) \tag{3}$$

Where α is the power transmission factor of passive component ($0 < \alpha < 1$) while G_1, G_2, ..., G_n and F_1, F_2, ..., F_n are gain and noise figure of individual amplifier respectively. An optimized high power comb source could provide carriers with maximum and minimum OSNR of $\sim$ 39 dB (P_{comb}= -12 dBm) and $\sim$ 35 dB (P_{comb}= -16 dBm) before data encoding via MZM (supplementary figure 3) indicating better OSNR can be achieved in comparison with current results. The variation in OSNR is due to the varying power of comb lines in $\sim$ 3 dB bandwidth. The variation in OSNR can be reduced and further improved by optimizing the ratio of κ_{ex} and κ_0 and second order dispersion.

IV. SUPPLEMENTARY NOTE 4: SCALABILITY

Increasing the number of nodes in the current system would require using soliton microcomb and AWG with lower FSR. Soliton microcombs operating at a repetition rate of 10 GHz have been demonstrated [11]. Similarly, InP based AWG with 100 $\times$ 10 GHz has been shown [12] indicating scaling feasibility in the future. In the current architecture, the SOAs scale with N^2 where N is the number of nodes if a single transmitter (MZM) is used. Here we propose to use multiple transmitters (MZM) on each node (figure 1 b, main text) to reduce N^2 scaling. This allows scaling the flat network without being limited by the low yield of large-scale InP devices. Detailed analysis on the scalability optical network is explained in ref [13].

V. SUPPLEMENTARY NOTE 5: FSR OF SOLITON MICROCOMB AND AWG

It is important to match the FSR of both soliton microcomb and AWG to standard ITU grid spacing for precise aligning of channels while the central channel can be aligned via thermal tuning. In the current demonstration, the FSR mismatch is $\sim$ 0.5 GHz (soliton microcomb), which can be further improved by using an optimized design that is more tolerant to fabrication errors [14] and improved fabrication [15]. InP-based AWG is a standard module that is designed (JEPPIX foundry) and fabricated at the commercial foundry (Fraunhofer HHI) providing a precise control over FSR.

SUPPLEMENTARY REFERENCES

* These authors contributed equally to this work.
† tobias.kippenberg@epfl.ch
‡ hitesh.ballani@microsoft.com

[1] P. Yin, J. R. Serafini, Z. Su, R.-J. Shiue, E. Timurdogan, M. L. Fanto, M. L. Fanto, and S. Preble, Optics Express **27**, 24188 (2019).
[2] J. Liu, A. S. Raja, M. H. P. Pfeiffer, C. Herkommer, H. Guo, M. Zervas, M. Geiselmann, and T. J. Kippenberg, Optics Letters **43**, 3200 (2018).
[3] B. Stern, X. Ji, Y. Okawachi, A. L. Gaeta, and M. Lipson, Nature **562**, 401 (2018).
[4] A. S. Raja, A. S. Voloshin, H. Guo, S. E. Agafonova, J. Liu, A. S. Gorodnitskiy, M. Karpov, N. G. Pavlov, E. Lucas, R. R. Galiev, A. E. Shitikov, J. D. Jost, M. L. Gorodetsky, and T. J. Kippenberg, Nature Communications **10**, 1 (2019).
[5] J. Liu, H. Tian, E. Lucas, A. S. Raja, G. Lihachev, R. N. Wang, J. He, T. Liu, M. H. Anderson, W. Weng, S. A. Bhave, and T. J. Kippenberg, Nature **583**, 385 (2020).
[6] C. Joshi, J. K. Jang, K. Luke, X. Ji, S. A. Miller, A. Klenner, Y. Okawachi, M. Lipson, and A. L. Gaeta, Optics letters **41**, 2565 (2016).
[7] P. Marin-Palomo, J. N. Kemal, M. Karpov, A. Kordts, J. Pfeifle, M. H. P. Pfeiffer, P. Trocha, S. Wolf, V. Brasch,

M. H. Anderson, R. Rosenberger, K. Vijayan, W. Freude, T. J. Kippenberg, and C. Koos, Nature **546**, 274 (2017).

[8] P. W. Juodawlkis, J. J. Plant, W. Loh, L. J. Missaggia, F. J. O'Donnell, D. C. Oakley, A. Napoleone, J. Klamkin, J. T. Gopinath, D. J. Ripin, S. Gee, P. J. Delfyett, and J. P. Donnelly, IEEE Journal of Selected Topics in Quantum Electronics **17**, 1698 (2011).

[9] Optilab LLC https://www.optilab.com/optical-amplifier.

[10] L. Schares, R. Budd, D. Kuchta, F. Doany, C. Schow, M. Möhrle, A. Sigmund, and W. Rehbein, in *2015 European Conference on Optical Communication (ECOC)* (2015) pp. 1–3.

[11] J. Liu, E. Lucas, A. S. Raja, J. He, J. Riemensberger, R. N. Wang, M. Karpov, H. Guo, R. Bouchand, and T. J. Kippenberg, Nature Photonics **14**, 486 (2020).

[12] F. M. Soares, N. K. Fontaine, R. P. Scott, J. H. Baek, X. Zhou, T. Su, S. Cheung, Y. Wang, C. Junesand, S. Lourdudoss, K. Y. Liou, R. A. Hamm, W. Wang, B. Patel, L. A. Gruezke, W. T. Tsang, J. P. Heritage, and S. J. B. Yoo, IEEE Photonics Journal **3**, 975 (2011).

[13] H. Ballani, P. Costa, R. Behrendt, D. Cletheroe, I. Haller, K. Jozwik, F. Karinou, S. Lange, K. Shi, B. Thomsen, and H. Williams, in *Proceedings of the Annual Conference of the ACM Special Interest Group on Data Communication on the Applications, Technologies, Architectures, and Protocols for Computer Communication*, SIGCOMM '20 (Association for Computing Machinery, New York, NY, USA, 2020) p. 782–797.

[14] B. Ouyang, Y. Xing, W. Bogaerts, and J. Caro, Opt. Express **27**, 38698 (2019).

[15] J. Liu, G. Huang, R. N. Wang, J. He, A. S. Raja, T. Liu, N. J. Engelsen, and T. J. Kippenberg, Nature communications **12**, 1 (2021).
